# The wildlife–livestock interface: a general perspective

**DOI:** 10.1093/af/vfad073

**Published:** 2024-02-14

**Authors:** Patricia Barroso, Christian Gortázar

**Affiliations:** Department of Veterinary Sciences, Faculty of Veterinary, University of Turin, Grugliasco, Turin, Italy; Department of Animal Health, Faculty of Veterinary, University of León, 24071 León, Spain; SaBio Instituto de Investigación en Recursos Cinegéticos (IREC) CSIC-UCLM-JCCM, 13071 Ciudad Real, Spain

Wildlife has historically coexisted with livestock in dynamic agroecosystems. However, the unceasing human population growth has given rise to an increase in animal protein demands. Consequently, livestock production has suffered an enormous expansion with changes in livestock breeding systems and a subsequent impact on biodiversity conservation. Livestock grazing is a widespread land use that occurs in a wide variety of ecosystems, occupying more than one-third of the earth’s land surface (Barroso and Gortázar, 2023; Cravino et al., 2023). These changes (farming practices, human demography, and land uses) together with changes in wildlife management and dynamics have led to increased interactions between wildlife and livestock across a variety of contexts. This is the origin of the concept ‘wildlife–livestock interface’. The wildlife–livestock interface is defined as a dynamic network of epidemiological and ecological connections between wildlife, livestock, and the physical space in which they overlap and potentially interact (Vicente et al., 2021). This interface changes along with natural landscapes and is vulnerable to human intervention (VerCauteren and Breck, 2023). This issue aims to bring together different aspects of wildlife–livestock coexistence, discussing the main positive and negative impacts on all the players implicated. To this end, six articles are compiled.

Barroso and Gortázar (2023) provide a global overview of the conflicts and opportunities at the wildlife–livestock interface and its multiple facets. The impacts of this coexistence on the environment can be positive or negative depending on the context (i.e., region, timeframe, farming system, and stocking rate). The main sources of conflict are shared pathogens, large predators and obligate scavengers, competition for resources, and fencing. All these conflicts must be addressed considering the interests of the relevant sectors. The interventions proposed comprise (i) zoning and land use planning, (ii) diversifying community livelihoods and lifting restrictions on wildlife harvest, (iii) establishing damage compensation and pasture fencing schemes, (iv) deploying biosafety measures to reduce wildlife–livestock contacts, and (v) manipulating livestock densities and wild herbivore populations through farm husbandry and hunting for a targeted use of chronic disturbance to improve ecosystem patterns and processes.

Cravino et al. (2023) and Barroso and Zanet (2023) focus on the bidirectional relationships between livestock production and biodiversity preservation and the practical combination of both concepts. These articles concur in suggesting that the protection of environmental and ecological resources and commercial livestock management should not be a contradiction.

Cravino et al. (2023) reflect on key factors that make the integration of livestock production with wildlife conservation possible (win–win outcomes). Livestock presence results in new environmental conditions (e.g., changes in vegetation structure or increased presence of dogs and humans) that can favor or hamper wildlife populations. In this article, the authors discuss those factors and scenarios which affect the positive or negative consequences of livestock breeding on wildlife. For example, if livestock production implies completely substituting native ecosystems for pasturelands (negative impact) or if it occurs on native grasslands or even partially modified savannas (minor impact on wildlife). Finally, Cravino et al. (2023) proposed several measures for livestock breeders to integrate livestock breeding and wildlife conservation while limiting habitat loss and fragmentation. Some of these measures consist of adjusting stocking rates to intermediate levels to avoid severe habitat degradation or opportunely rotating the herd between paddocks to generate heterogeneous landscapes.

Barroso and Zanet (2023) expose the results of the livestock–biodiversity relationship of a pilot monitoring study in mainland Spain. These results suggest a positive relationship between livestock presence and biodiversity indexes since areas with livestock showed higher species richness, biodiversity, and relative abundance of rodents compared to those free of livestock. In this context, they highlight the importance of facilitating extensive livestock breeding and incentivizing landowners for biodiversity-cognizant livestock production instead of over-regulating livestock-based land use, thereby encouraging their active involvement in biodiversity safeguarding.

VerCauteren and Breck (2023) pinpoint and broadly categorize the main conflicts derived from the wildlife–livestock interface into three groups: competition for resources, those related to disease transmission, and predation. They propose mitigation measures for the management of livestock and wildlife separately but highlight that a dynamic combination of both groups of actions could become the more successful strategy for the final goal of reducing conflicts and preserving biodiversity, improving the harmony of the wildlife–livestock interface, preserving wildlife conservation, and fulfilling livestock needs. However, they mention that breakdowns are common even using an integrated approach. Thus, they recommend an increased emphasis on proactive prevention and management of wildlife–livestock conflicts on both the wildlife and livestock sides.

Karmacharya et al. (2023) and Treves et al. (2023) elaborate on specific conflicts of the wildlife–livestock interface: shared pathogens and large predators. Karmacharya et al. (2023) reflect on the importance of shared infections at this interface, which exerts important consequences for public health, economy (causing over 20% of global livestock losses), and biodiversity. The spill-over and transmission dynamics of shared infections are multifactorial and occur in complex multihost communities. Changes in pathogen distribution and maintenance are expected at different interfaces in the context of the ongoing global crisis (Karmacharya et al., 2023). Measures proposed by the authors include (i) enhanced disease surveillance systems, (ii) implementing effective risk mitigation and management strategies, and (iii) fostering coordination and collaboration among national, regional, and global government and non-government agencies and stakeholders.

Treves et al. (2023) explore different methods to evaluate the effectiveness of nonlethal methods (randomized, controlled trials and gold standard) to reduce a specific conflict of the wildlife–livestock interface: large carnivore attacks on livestock. They summarize lessons learned from their application in different geographical regions and regarding a broad range of target livestock species. Their review suggests that nonlethal methods can result functionally in preventing carnivore approaches and attacks on farms and achieve the elusive triple-win for wildlife, domestic animals, and livelihoods.

Designing agroecosystems for optimizing and balancing wildlife conservation and livestock production in the long term is a challenging necessity to reach sustainable rural environments. In this regard, cooperation among landowners, farmers, conservationists, and government agencies is essential. Our capability to manage wildlife conservation in areas with livestock presence, including protected areas and rangelands, is influenced by our knowledge of its bidirectional consequences. Thus, research and multidisciplinary approaches under the One Health framework are needed to maximize food safety, global health, and ecosystem services while minimizing the potential negative consequences of the wildlife–livestock interface.

Funding

This study is a contribution to the projects: (1) “Ganadería con una sola salud: monitoreo ambiental y mitigación de riesgos para la producción ganadera segura y sostenible y la conservación de la biodiversidad” grant PLEC2021-008113 funded by MCIN/AEI/10.13039/501100011033 and by the European Union NextGenerationEU/PRTR; and (2) “Biodiversity, health and sustainability of extensive ruminant grazing in Spain” (BIO-GRAZ) grants PID2022-141906OB-C21 and PID2022-141906OB-C22 funded by MCIN/AEI/10.13039/501100011033 and by ERDF A way of making Europe. P.B. was supported by the EU-NextGenerationEU funds through the 2022-2024 Margarita Salas call for the requalification of the Spanish University System, convened by the University of Castilla-La Mancha.

## About the Authors

**P. Barroso** is a researcher for the Department of Veterinary Sciences of the University of Turin. Main research activities include health and population monitoring of wildlife, eco-epidemiology of shared pathogens at the wildlife–livestock–human interface, and disease control. Main research topics include the long-term dynamics and epidemiology of shared pathogens in complex host communities, the design of integrated wildlife monitoring systems and the study of biodiversity–disease relationships.

**C. Gortázar** heads the Health and Biotechnology (SaBio) group at the National Wildlife Research Institute IREC (Universidad de Castilla La Mancha and CSIC) in Ciudad Real, Spain. His main research activities combine ecology, veterinary sciences, and biotechnology to address global challenges in wildlife management and shared infection control at the wildlife–livestock interface.
